# Age and an obesogenic diet affect mouse behaviour in a sex‐dependent manner

**DOI:** 10.1111/ejn.16070

**Published:** 2023-06-28

**Authors:** Emily J. Mort, Surina Fordington, Sophie Heritage, Abigail L. Fowden, Susan Jones, Emily J. Camm

**Affiliations:** ^1^ Department of Physiology, Development and Neuroscience University of Cambridge Cambridge UK; ^2^ The Ritchie Centre Hudson Institute of Medical Research Clayton Victoria Australia

**Keywords:** age differences, elevated plus maze, high fat–high sugar diet, open field, sex differences, social preference

## Abstract

Obesity is rising globally and is associated with neurodevelopmental and psychiatric disorders among children, adolescents and young adults. Whether obesity is the cause or the consequence of these disorders remains unclear. To examine the behavioural effects of obesity systematically, locomotion, anxiety and social behaviour were assessed in male and female C57Bl/6J mice using the open field, elevated plus maze and social preference task. First, the effects of age and sex were examined in control mice, before investigating post‐weaning consumption of a high fat–high sugar diet commonly consumed in human populations with high rates of obesity. In the open field and elevated plus maze, locomotor activity and anxiety‐related behaviours reduced with aging in both sexes, but with different sex‐specific profiles. The high fat–high sugar diet reduced food and calorie intake and increased body mass and fat deposition in both sexes. In the open field, both male and female mice on the obesogenic diet showed reduced locomotion; whereas, in the elevated plus maze, only females fed with the obesogenic diet displayed reduced anxiety‐related behaviours. Both male and female mice on the obesogenic diet had a significantly higher social preference index than the control group. In conclusion, the findings demonstrate that the behavioural effects of age and diet‐induced obesity all depend on the sex of the mouse. This emphasises the importance of considering the age of the animal and including both sexes when assessing behavioural phenotypes arising from dietary manipulations.

## Graphical abstract

Mouse locomotor and anxiety behaviours were changed across the age range of 8–22 weeks, with the two sexes adopting different behavioural strategies in coping with novel, stressful conditions. Feeding a high fat–high sugar (HFHS) diet from weaning also altered anxiety, locomotion and social preference in adult male and female mice. This work highlights the importance of age and sex in behavioural studies.

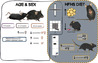

**Abbreviations**
Elevated plus maze: (EPM)
High fat–high sugar: (HFHS)
Open field: (OF)
Social preference: (SP)

## 1 INTRODUCTION

The incidence of obesity defined by the World Health Organisation as a body mass index (BMI) of greater than 30 kg (weight)/m^2^ (height) is increasing rapidly worldwide in both developed and developing countries, largely as a result of dietary changes (World Health Organisation, 2021). In the UK, the incidence of obesity has tripled in the last 50 years, with about two‐thirds of the adult population now classified as overweight or obese, although the exact figures vary with age and ethnicity (NHS Digital, 2020). Obesity is also increasing in children, with approximately 20% of 10–11 year olds classed as obese in 2018 (NHS Digital, 2020). Obesity and being overweight (BMI 25–29.9 kg/m^2^) are known to be major risk factors for adult cardiovascular and metabolic diseases (Powell‐Wiley et al., 2021). They also have a negative impact on behaviour and mental health from childhood onwards, with associations between a high BMI and a wide range of behavioural alterations in human epidemiological studies, including depression, impaired memory and poorer cognitive performance (Edlow, 2017). In particular, obesity is associated with increased anxiety (Fulton et al., 2022) and risk‐taking behaviours in both adolescent and adult human populations (Ratcliff et al., 2011). The specific alterations in behaviour have also been shown to be linked to age and sex in certain instances. However, whether obesity is the direct cause or the consequence of the behavioural changes remains unclear, as the prevalence of anxiety disorders increases with obesity, while anxiety and risk‐taking behaviours predispose individuals to weight gain.

The bidirectional nature of this relationship can be studied more systematically in experimental animals like rodents, as dietary intake and adiposity can be measured precisely in these species. In rodents, obesogenic diets have been shown to both increase and decrease anxiety and risk‐taking behaviours, which may reflect differences in the age of the mice and/or in the dietary regime between the studies (Clark, Crean, & Senior, 2022; Clark, Reichelt, et al., 2022; Haleem & Mahmood, 2021; Lopez‐Taboada et al., 2020; Yoshizaki et al., 2020). Often, little information is provided on the dietary composition, specific nutrient and calorie consumption or on the actual degree of adiposity, as body weight is frequently used as a proxy measure of obesity in rodents. Many of the studies have used high fat diets rather than diets high in both fat and sugar, more commonly eaten by increasingly overweight and obese human populations. In addition, the majority of behavioural studies of obesogenic diets in rodents have focussed on males. Because of the possibility of greater variability in female data during the oestrus cycle, few studies have investigated the behavioural consequences of obesogenic diets in female mice. Even fewer have studied both males and females simultaneously using the same dietary and experimental regime. These omissions make it difficult to determine if the behavioural changes seen in previous studies of calorie‐dense diets in mice are attributable to obesity per se or reflect other dietary factors known to alter behaviour such as protein deficiency (Clark, Reichelt, et al., 2022; Lopez‐Taboada et al., 2020).

Moreover, although many studies have investigated changes in behaviour and cognition with age over narrow windows of time, some have employed large age ranges between experimental groups (e.g., several months; Lalonde & Strazielle, 2009) or classified a large age range as one experimental cohort (e.g., 8–12 months; Shoji et al., 2016). Levels of anxiety‐related and social behaviour have been shown to change between adolescence and adulthood when assessed using common tasks such as the open field (OF), elevated plus maze (EPM) and social preference (SP) tasks (Lalonde & Strazielle, 2009; Macri et al., 2003; Nolte et al., 2019; Shoji et al., 2016). Consequently, the pattern of behavioural change with age requires further investigation even on a standard rodent diet.

The first aim of the current study was to assess behavioural changes associated with aging and sex in mice to provide baseline data (or ‘provide a baseline’) before investigating the effects of an obesogenic diet. Gross locomotion and anxiety‐like and social behaviours were evaluated in male and female C57Bl/6J mice using the OF, EPM and SP tasks. The OF and EPM tasks were carried out at three key stages of the life course—the boundary between late adolescence and early adulthood (8–9 weeks), early adulthood (11–13 weeks) and middle adulthood (20–22 weeks). The age range within the groups was kept narrow to obtain precise behavioural measures at each life stage. At 20–22 weeks, the mice are still in good health; thus, any behavioural changes up to this age should reflect normal aging and not pathological processes. The second and the main aim of the study was to investigate the consequences of consuming a bespoke high fat–high sugar (HFHS) diet on locomotor, anxiety and social behaviours in both male and female mice at 11–13 weeks of age in relation to their calorie and protein intake, somatic growth and fat deposition.

## 2 METHODS

### 2.1 Experimental design

All animal experimentation was carried out under the UK Home Office Animals (Scientific Procedures) Act 1986, following the ethical review by the University of Cambridge (Item 7; 2018 | University Biomedical Services [UBS] [cam.ac.uk]). A total of 186 C57Bl/6J mice were used in this study. The initial animals were purchased commercially (Charles River, Margate, UK) but, thereafter, the mice were bred in‐house. They were group housed by sex (*n* = 2–5 mice per cage, for the duration of the study) under a 12:12 h dark/light photocycle with ad libitum access to food and water. The majority of the animals studied (*n* = 136) were fed a standard rodent diet (RM3, Special Dietary Services [SDS], Witham, UK; 11%kcal fat, 62%kcal carbohydrate of which 7%kcal is simple sugar, 27%kcal protein and a water content of 10%). The remainder were fed a customised HFHS diet from weaning at 21 days of postnatal age to induce increased adiposity in adulthood. The HFHS diet was made by combining high‐fat diet pellets (D12451 diet, Research Diets Inc., Lynge, Denmark) with condensed milk (Carnation, Nestle, Gatwick, UK) and water to form patties that were baked at 55°C for 46–48 h, as described previously (Napso et al., 2022). The final nutritional composition of the HFHS diet was 38%kcal fat, 45%kcal carbohydrate of which 33%kcal was simple sugars, 17%kcal protein, and a water content of 12%. The HFHS and standard diets were replenished every 48 h during the period of the study to ensure palatability.

In order to assess the effects of age and sex on behavioural outcomes, groups of mice (primarily non‐siblings, purchased from Charles River) fed the standard diet were examined at three different ages: 8–9 (*n* = 13 females, *n* = 13 males), 11–13 (*n* = 12 females, *n* = 15 males) and 20–22 weeks (*n* = 5 females, *n* = 18 males). At the end of the experimental period, the mice used to assess the effects of age and sex on behavioural outcomes were killed by cervical dislocation.

To determine the impact of an obesogenic diet on mouse behaviour, male and female mice were fed the HFHS diet (*n* = 26 females, *n* = 25 males) for 8–9 weeks before the beginning of behavioural testing at 11–13 weeks and were compared with a control cohort of 11–13 week old mice fed the standard chow (*n* = 36 females, *n* = 31 males). To measure calorie and protein intake, food intake on the two diets was measured weekly over a 24 h‐period from Week 9 to 13 for a subset of the cages and expressed as intake per day per gram of the total weight of mice in each cage. The number of mice per cage was not significantly different for the two sexes and diets (*P* = 0.90, Welch's ANOVA). Body weight of the individual mice was also monitored weekly from weaning to the end of the experimental period to measure their weight gain. After finishing the behavioural testing at 14 weeks, a subset of the females and males in the two groups were killed by administration of a lethal dose of anaesthetic (20 mg/10 g, Merial‐Euthatal, Coventrus, Dumfries, UK) to minimise potential tissue trauma (Control, *n* = 20 females; *n* = 15 males; HFHS, *n* = 13 females, *n* = 13 males). These mice were used to quantify total body fat and lean mass by Dual Energy X‐ray Absorptiometry (DEXA) scanning (Lunar PIXImus densitometer; GE Healthcare, Madison, WI, USA) and/or to measure the weights of the individual fat deposits and body organs. The remainder of the mice in the dietary study were killed by cervical dislocation.

### 2.2 Behavioural testing

The majority of the mice carried out two different behavioural tests at intervals of 3–4 days. To study the effects of age and sex, the two tests used were the OF and EPM tasks. For the dietary comparison, the behavioural tests used were the EPM or one of the following combinations of tasks: the OF and EPM or the OF and SP task. The mice were randomly assigned to the behavioural tasks; no siblings of the same sex were used in any set of behaviour tests. A ceiling‐mounted webcam was used to record each testing session, with the recordings analysed manually blinded to the cohort where possible. Female mice were tested in proestrus to account for the effect of oestrus cycle on behaviour (Gangitano et al., 2009; Marcondes et al., 2001). Oestrus cycle stage was determined by visual inspection to avoid invasive vaginal smears, which could have stressed the mice before the assessment. Oestrus cycle status was not assessed at post‐mortem.

### 2.3 OF

The OF test assesses novel environment exploration and general locomotor activity and provides an initial screen for anxiety‐related behaviour in rodents (Prut & Belzung, 2003). The OF arena comprised a 50cm^3^ box with 16 5 × 5cm grids on the bottom, allowing for the measurement of movement. The box was separated into two zones: the periphery and the four grids in the centre, to allow the assessment of thigmotaxis, the natural tendency of rodents to stay close to walls when traversing an open environment. Mice were placed in the centre of the box and allowed to freely explore the arena for 10 min. If a mouse crossed at the point where two lines intersected, such as the corner of one of the grid squares, it was only counted as one line. A mouse was considered to have entered the central zone once all four paws had crossed the boundary; as soon as any paws entered the periphery, the mouse was no longer counted as being within the centre. The number of floor grid lines crossed was used as a measure of locomotion. The time spent in the centre and periphery and the total number of rears were also quantified as a measure of exploration and anxiety (Sturman et al., 2018).

### 2.4 EPM

The EPM is a widely used behavioural assay for rodents that has been validated to assess anxiety, locomotion, exploration and risk‐taking behaviours (Walf & Frye, 2007). It is considered the gold standard method for the evaluation of anxious‐type behaviour in rodents. The EPM used in the current study consisted of 65 cm long open and closed arms fixed in a plus‐formation, with 15 cm high walls. The maze was elevated 52 cm from the ground. Each mouse was placed in one end of the closed arm facing the wall and was allowed to freely explore the maze for 5 min, after which it was returned to its home cage. A mouse was considered as having entered an area when all four paws had crossed the boundary and was classified as having left that area as soon as any paw crossed back over the boundary. The following measures were quantified: open arm time, number of full entries to the open and closed arm and number of times the mouse reached the end of the open arm (end open arm exploration). The total number of vertical rears was also quantified.

### 2.5 SP

The SP task (Moy et al., 2004) comprises a three‐chambered box, with containers placed in the two outer chambers that can house mice. Test mice were habituated to the three‐chambered arena for 15 min. Empty containers were then added to the arena, and the mice were allowed to explore further for 30 min, thereby removing the novelty of the environment so that the focus remained on the contents of the container, rather than the containers themselves driving the exploration. The SP test was commenced after the habituation period. The position of the containers was balanced between the diet groups. After 5 min of habituation for the unfamiliar sex‐ and strain‐matched mouse (intruder), the test mouse was placed into the middle chamber and allowed to freely explore for 10 min. Interaction time with the intruder (social stimulus, TS) and non‐social stimulus (TNS) was quantified. The SP index was calculated using the following formula:

$$\mathrm{SP}\;\text{Index}=\left(\mathrm{TS}-\mathrm{TNS}\right)/\left(\mathrm{TS}+\mathrm{TNS}\right)$$

### 2.6 Statistical analysis

All statistical analyses were performed using GraphPad Prism (version 9.4.1 for Windows, GraphPad Software, San Diego, CA, USA). A Shapiro–Wilk normality test was used to examine if the data were normally distributed. Mice were deemed to be statistical outliers and excluded if their mean was more than two standard errors from the group mean in three or more measures. Data are presented as mean ± standard error of the mean (SEM). A *t*‐test or Mann–Whitney non‐parametric test was used, as appropriate, to compare biometric and behavioural measures between two groups (control versus HFHS). A one‐way ANOVA followed by a Dunnett's post hoc test or Kruskal–Wallis with Dunn's post hoc test was used to compare the effects of age (8–9, 11–13 and 20–22 weeks of age) on behavioural outcome measures. A mixed effects analysis or two‐way ANOVA followed by a Sidak's post hoc test was used to compare the effects of treatment (between‐subject factor) and age (within‐subject factor) on food intake and body mass; sphericity was not assumed in any test. Statistical significance was accepted as *P* ≤ 0.05.

## 3 RESULTS

### 3.1 Effect of age and sex on locomotor and anxiety‐related behaviour

**FIGURE 1:**
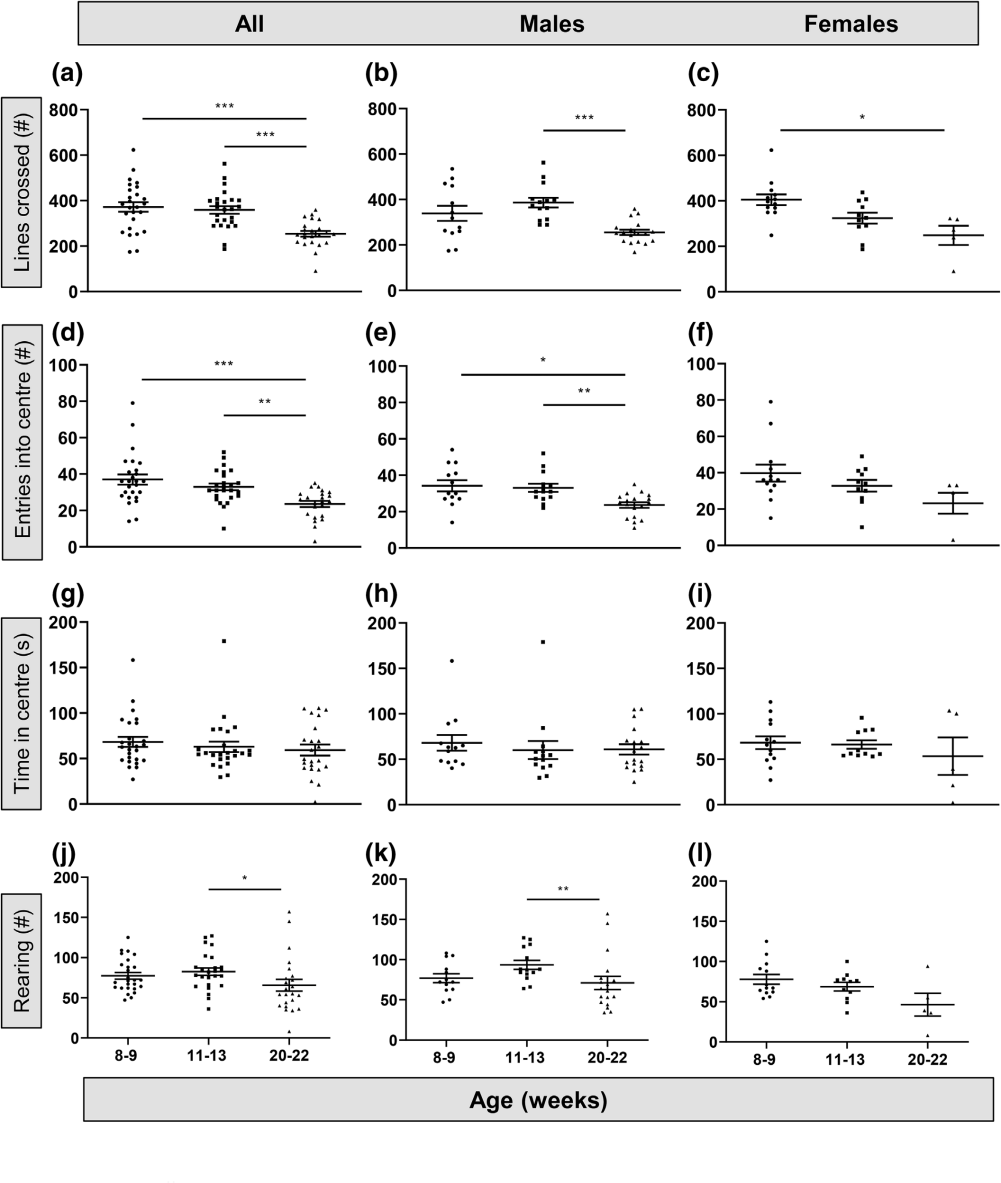
Age and sex differences in mouse behaviour in the open field (OF). (a) Locomotor activity in the OF measured as the number of lines crossed for all mice tested at either 8–9, 11–13 or 20–22 weeks (*P* < 0.0001, ANOVA). (b) Effect of age on the number of lines crossed in male mice (*P* < 0.0001, ANOVA). (c) Number of lines crossed in female mice (*P* = 0.02, ANOVA). (d) Open space anxiety‐related behaviour in the OF measured as the number of entries into the centre for all mice tested at either 8–9, 11–13 or 20–22 weeks (*P* = 0.0001, Kruskal–Wallis). (e) Number of entries into the centre in male mice (*P* = 0.002, ANOVA). (f) Number of entries into the centre in female mice (*P* = 0.08, Kruskal–Wallis). (g) Time (seconds) spent in the centre of the OF for all mice tested at either 8–9, 11–13 or 20–22 weeks (*P* = 0.46, Kruskal–Wallis). (h,i) There were no significant age effects in male (*P* = 0.50, Kruskal–Wallis) or female (*P* = 0.72, Kruskal–Wallis) mice. (j) Exploratory rearing in the OF in all mice tested at either 8–9, 11–13 or 20–22 weeks (*P* = 0.017, Kruskal–Wallis). (k) There was a significant effect of age on the number of rears in male mice (*P* = 0.01, Kruskal–Wallis). (l) There was no effect of age in female mice (*P* = 0.17, ANOVA). Numbers of mice (see Methods): 8–9 weeks, 13 males and 13 females; 11–13 weeks, 14 males and 11 females; 20–22 weeks, 18 males and five females. Post‐hoc tests (see Methods): *P < 0.05; **P < 0.001; ***P < 0.0001.

In the OF arena, both age and sex affected locomotion and anxiety‐related behaviour over the period from 8 to 22 weeks of age (Figure 1). Specifically, the age of mice at testing had a significant effect on the number of lines crossed in the OF, an index of locomotion (Figure 1a; *F*_2,46_ = 18.27, *P* < 0.0001, Welch's ANOVA). With increasing age, there was a reduction in the number of lines crossed in both male (Figure 1b; *F*_2,22_ = 15.04, *P* < 0.0001, Welch's ANOVA) and female mice (Figure 1c; *F*_2,11_ = 5.74, *P* = 0.02, Welch's ANOVA), although with a different age profile in each sex, with males showing a decrease in lines crossed between 11–13 and 20–22 weeks while females showed a decrease in lines crossed from 8–9 to 20–22 weeks (Figure 1b,c). The number of entries into the centre of the OF, the most anxiogenic area of the arena (Figure 1d,e), decreased significantly with age in the males (Figure 1e; *F*_2,24_ = 8.4, *P* = 0.002, Welch's ANOVA), but not in the females (Figure 1f; *P* = 0.08, Kruskal–Wallis). Age had no effect on the time spent in the centre (Figure 1g–i) in either sex (males, *P* = 0.50, Kruskal–Wallis; females, *P* = 0.72, Kruskal–Wallis). Total rearing in the OF (Figure 1j–l) decreased significantly with age in male (*P* = 0.01, Kruskal–Wallis) but not in female (*P* = 0.17, Welch's ANOVA) mice. Overall, the data suggest that age of testing influenced locomotor activity in both sexes but affected anxiety‐related behaviour and rearing only in males. These findings demonstrate clear sex‐specific differences in behaviour in the OF between 8 and 22 weeks of age.

**FIGURE 2:**
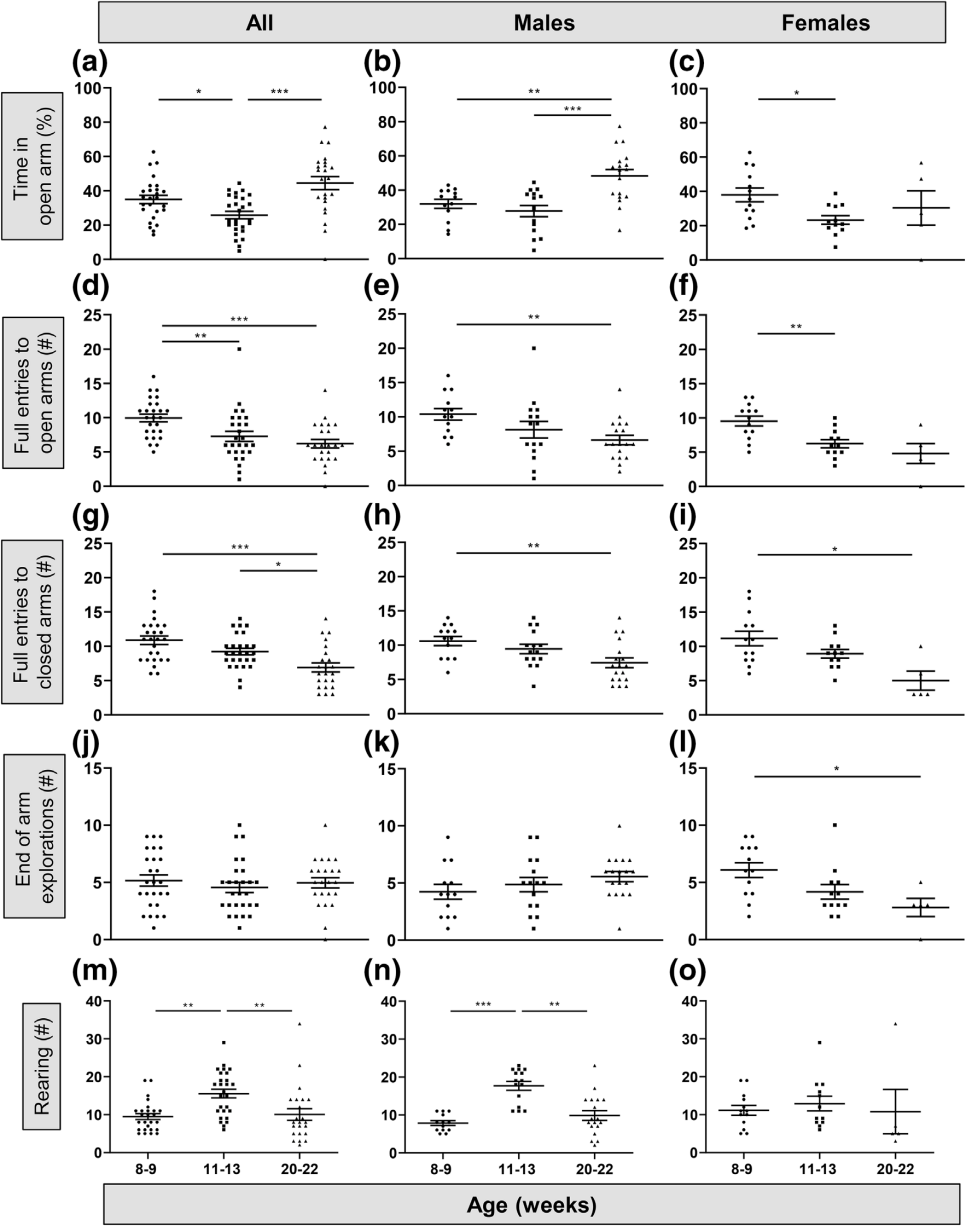
Age and sex differences in mouse behaviour in the elevated plus maze (EPM). (a) Height anxiety‐related behaviour in the EPM measured as percentage time spent in the open arm for all mice tested at either 8–9, 11–13 or 20–22 weeks (*P* = 0.0001, ANOVA). (b) Percentage time in the open arm in male mice (*P* = 0.0006, ANOVA). (c) Percentage time in the open arm in female mice (*P* = 0.04, ANOVA). (d) Full entries into the open arm of the EPM, a measure of both anxiety and locomotion, was tested in all mice at either 8–9, 11–13 or 20–22 weeks (*P* = 0.0001, Kruskal–Wallis). (e) Full entries to the open arm in male mice (*P* = 0.008, ANOVA). (f) Full entries to the open arm in female mice (*P* = 0.01, ANOVA). (g) Full entries into the closed arm, a measure of locomotion in the EPM, for all mice tested at either 8–9, 11–13 or 20–22 weeks (*P* = 0.0003, ANOVA). (h) Full entries into the closed arm in male mice (*P* = 0.01, ANOVA). (i) Full entries into the closed arm in female mice (*P* < 0.02 Kruskal–Wallis). (j) Explorations to the end of the open arm for all mice tested at either 8–9, 11–13 or 20–22 weeks (*P* = 0.65, Kruskal–Wallis). (k) End of open arm explorations in male mice (*P* = 0.25, ANOVA). (l) End of open arm explorations in female mice (*P* = 0.03, Kruskal–Wallis). (m) Total rearing for all mice tested at either 8–9, 11–13 or 20–22 weeks (*P* = 0.0002, Kruskal–Wallis). (n) Rearing in male mice (*P* < 0.0001, Kruskal–Wallis). (o) Rearing in female mice (*P* = 0.21, Kruskal–Wallis). Numbers of mice (see Methods): 8–9 weeks, 13 males and 13 females; 11–13 weeks, 15 males and 12 females; 20–22 weeks, 18 males and five females. Post‐hoc tests (see Methods): *P < 0.05; **P < 0.001; ***P < 0.0001.

Age and sex were also significant influences on behaviour in the EPM between 8 and 22 weeks of age (Figure 2). With both sexes combined, there was a significant effect of age on the percentage of time spent in the open arm of the maze, with significantly less time (percentage of the total testing time) in the open arm at 11–13 weeks than at the other two ages (Figure 2a; *F*_2,45_ = 10.19, *P* = 0.0001, Welch's ANOVA). This suggests that anxiety in the EPM was maximal at 11–13 weeks. However, the overall decrease in the percentage of time spent in the open arm between 8–9 and 11–13 weeks of age was primarily due to the females (Figure 2c; *F*_2,9.5_ = 4.5, *P* = 0.04, Welch's ANOVA), as there was no change in this parameter in the males between these ages (Figure 2b). In contrast, the percentage of time spent in the open arm was significantly greater at 20–22 weeks of age than at the earlier ages in male mice but was not significantly different at 20–22 weeks relative to the younger ages in the females. By analysing the sexes separately, these data reveal a sex‐specific difference in anxiety‐related behaviour within the EPM with age; female mice show increased anxiety by 11–13 weeks of age, while male mice show less anxiety by 20–22 weeks of age.

With increasing age, the number of full entries into either the closed or open arms of the EPM (Figure 2d–i) decreased in both male and female mice, suggesting that both sexes became more stationary whether in the centre or in the closed or open arms as they aged (males: Figure 2e, open arm; *F*_2,26_ = 5.84, *P* = 0.008; Figure 2h, closed arm; *F*_2,28_ = 5.1, *P* = 0.01; females: Figure 2f, open arm; *F*_2,10_ = 7.37, *P* = 0.01; Figure 2i, closed arm; *P* = 0.017; all Welch's ANOVA except female closed arm, Kruskal–Wallis). In addition, there was a significant decrease in the number of explorations to the end of the open arm in the female (Figure 2l; *P* = 0.025, Kruskal–Wallis) but not in the male (Figure 2k; *F*_2,26_ = 1.44, *P* = 0.25, Welch's ANOVA) mice with aging. Furthermore, total rearing in the EPM was age and sex‐related (Figure 2m–o). In the males, rearing increased between 8–9 and 11–13 weeks of age followed by a significant decrease between the ages of 11–13 and 20–22 weeks (Figure 2n; *P* < 0.0001, Kruskal–Wallis). Conversely, there was no effect of age on rearing in female mice (Figure 2o, *P* = 0.21, Kruskal–Wallis). Overall, the data suggest that both males and females show less locomotor activity with age, with female mice showing increased anxiety‐related behaviour with age; whereas, males showed less or no change in anxiety‐related behaviour and an increase in total rearing at 11–13 weeks of age.

### 3.2 Food intake, growth rate and fat deposition on a HFHS diet

**FIGURE 3:**
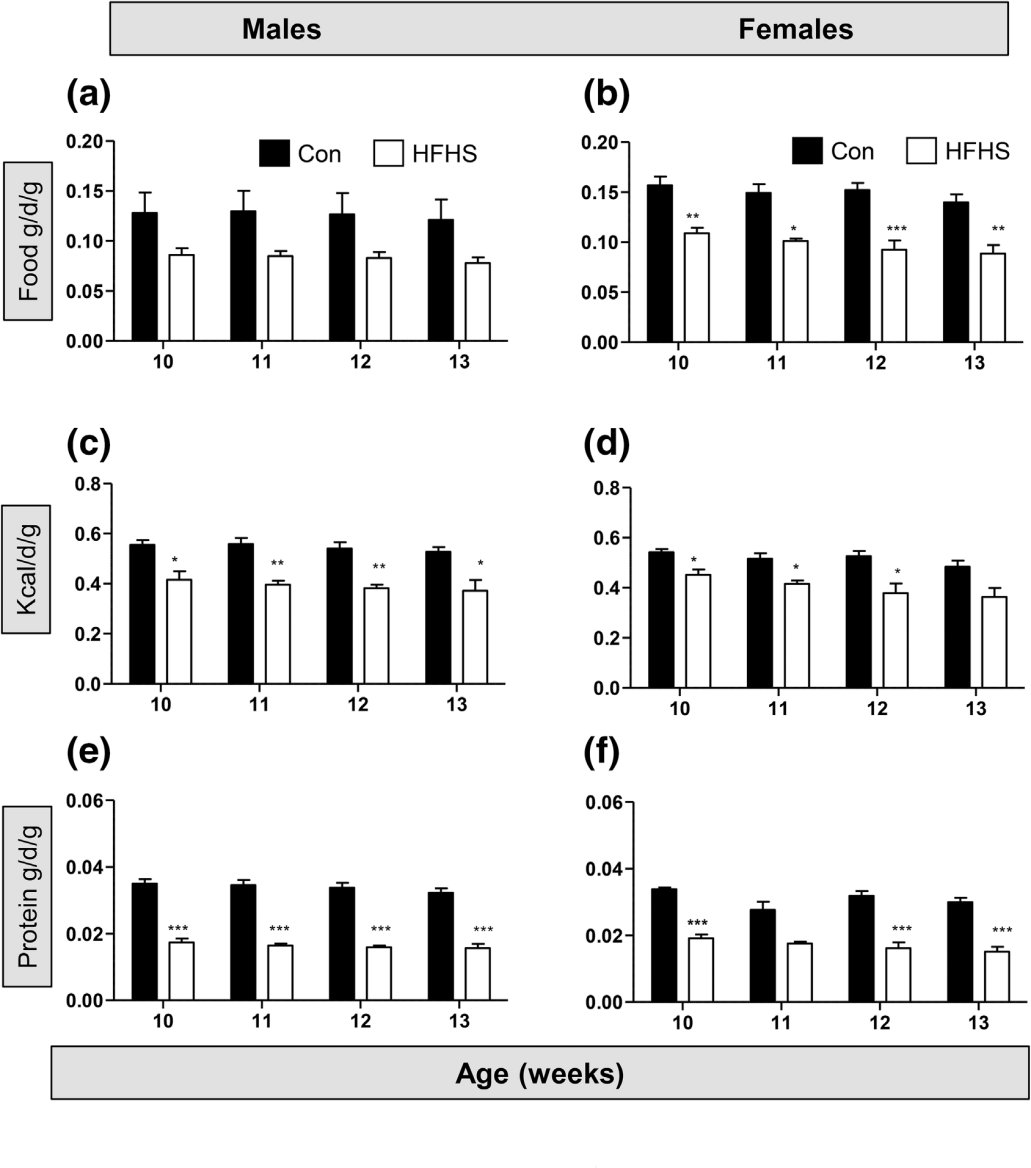
An obesogenic diet alters nutrient intake in male and female mice. (a) Food intake (gram of food per day per gram of mouse) in male mice fed either a control diet (filled bars) or a high fat–high sugar (HFHS) diet (open bars) from weaning, measured between 10 and 13 weeks. (b) Food intake (gram of food per day per gram of mouse) in female mice fed either a control diet or a HFHS diet from weaning, measured between 10 and 13 weeks. Two‐way ANOVA; posthoc test of diet effect: *P < 0.05; **P < 0.01; ***P < 0.001. (c) Kilocalorie intake (kcal per day per gram of mouse) in male mice fed either a control diet or a HFHS diet from weaning, measured between 10 and 13 weeks. Mixed effects analysis; posthoc test of diet effect: *P < 0.05; **P < 0.01. (d) Kilocalorie intake (kcal per day per gram of mouse) in female mice fed either a control diet or a HFHS diet from weaning, measured between 10 and 13 weeks. Two‐way ANOVA; posthoc test of diet effect: *P < 0.05. (e) Protein intake (gram of protein per day per gram of mouse) in male mice fed either a control diet or a HFHS diet from weaning, measured between 10 and 13 weeks. Mixed effects analysis; posthoc test of diet effect: ***P < 0.001. (f) Protein intake (gram of protein per day per gram of mouse) in female mice fed either a control diet or a HFHS diet from weaning, measured between 10 and 13 weeks. Two‐way ANOVA; posthoc test of diet effect, ***P < 0.001. Numbers of cages of mice: control diet, five cages of males, five cages of females; HFHS diet, seven to eight cages of males, 10 cages of females.

In order to determine the effect of increased adiposity on locomotion and on anxiety‐related and social behaviours of male and female mice, they were fed a HFHS diet from weaning. A mixed effects analysis was performed to compare the effects of age and diet on food, kilocalorie and protein intake between 10 and 13 weeks. In male mice (Figure 3a), there was a main effect of diet (*P* = 0.03) but not of age (*P* = 0.13) on food intake, with no interaction between age and diet (*F*_3,32_ = 0.15, *P* = 0.93). In female mice (Figure 3b), there was also a main effect of diet (*P* < 0.0001) but not of age (*P* = 0.15), with no interaction between these two factors in influencing food intake (*F*_3,39_ = 0.24, *P* = 0.87). For kilocalorie intake of the male mice (kcal per day per gram of mouse), there was a main effect of diet (*P* = 0.0007) but not of age (*P* = 0.21), with no interaction of both factors (Figure 3c; *F*_3,29_ = 0.18, *P* = 0.90). For female mice (Figure 3d), kilocalorie intake per day per gram of mouse varied significantly with diet (*P* < 0.0001), with no interaction between age and diet (Figure 5d; *F*_3,39_ = 0.31, *P* = 0.80). There was also a main effect of diet (*P* < 0.0001) on protein intake (grams per day per gram of mouse) in male mice, with no significant interaction between age and diet (Figure 3e, *F*_3,32_ = 0.36, *P* = 0.78). In females, there was a main effect of diet (*P* < 0.0001) and age (*P* = 0.049), but no significant interaction between the two factors (Figure 3f; *F*_3,39_ = 1.47, *P* = 0.23).

**FIGURE 4:**
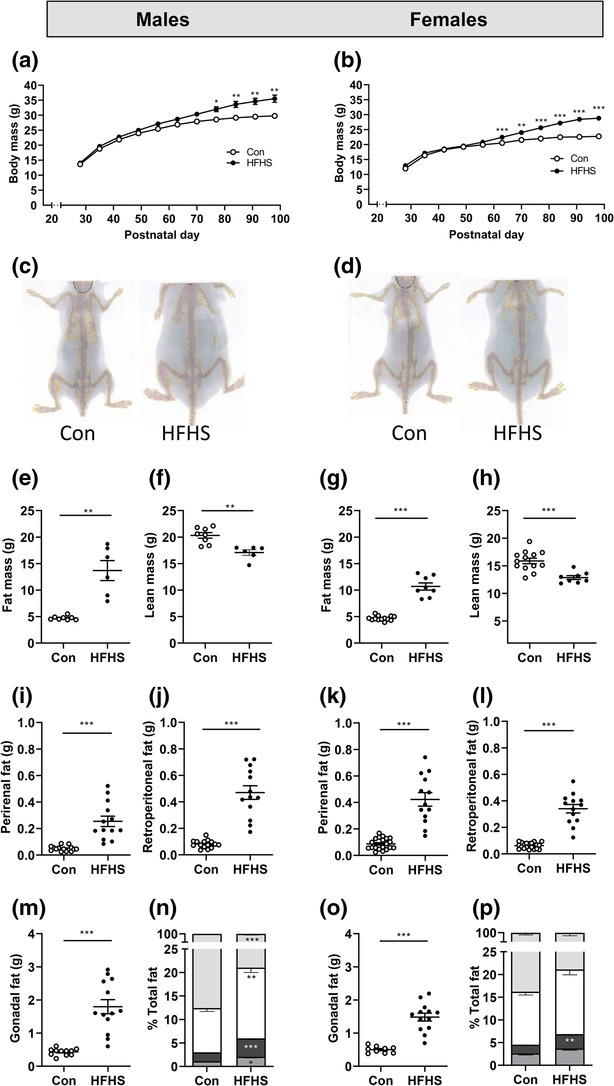
An obesogenic diet affects growth and adiposity in male and female mice. (a) Body mass (g) of male mice on either a control (*n* = 14–19) or high fat–high sugar (HFHS) (*n* = 13–14) diet from weaning until Postnatal day 98. Mixed effects analysis; post hoc test of diet effect: *P < 0.05; **P < 0.01. (b) Body mass (g) of female mice on either a control (*n* = 16–18) or HFHS (*n* = 14) diet from weaning until Postnatal day 98. Post hoc test of diet effect: **P < 0.01; ***P < 0.001. (c) Example dual energy X‐ray absorptiometry (DEXA) scans of male mice aged 98 days on the control diet (left) or HFHS diet (right). (d) Example DEXA scans of female mice aged 98 days on the control diet (left) or HFHS diet (right). (e) Fat mass (g) and (f) lean mass (g) of male mice on the control (*n* = 8) or HFHS (*n* = 6) diet, determined by DEXA (**P = 0.005, *t*‐test; *P* = 0.001, *t*‐test). (g) Fat mass (g) and (h) lean mass (g) of female mice on the control (n = 13) or HFHS (n = 8) diet, determined by DEXA (***P < 0.001, *t*‐test). Post‐mortem measurements of organ fat deposits following a control or HFHS diet: (i) Perirenal fat in male mice (***P = 0.0002, *t*‐test; *n* = 13–15). (j) Retroperitoneal fat in male mice (***P < 0.0001, *t*‐test; *n* = 13–15). (k) Perirenal fat in female mice (***P < 0.0001, *t*‐test; *n* = 13–20). (l) Retroperitoneal fat in female mice (***P < 0.0001, *t*‐test; *n* = 13–20). (m,o) Gonadal fat in male (***P < 0.0001, *t*‐test; *n* = 11–13) and female mice (***P < 0.0001, *t*‐test; *n* = 9–13). (n) Organs fat expressed as a percentage of total fat measured post‐mortem in male mice (perirenal, mid‐grey; retroperitoneal, dark grey; gonadal, white; other, pale grey). (p) Organs fat expressed as a percentage of total fat measured post‐mortem in female mice (colour code as for male mice in [n]). Data in (n) and (p) were arcsin transformed for statistical analysis of fat deposits in HFHS versus control diet (perirenal fat males, *P* = 0.015, females, *P* = 0.08; gonadal fat males, *P* = 0.002, females, *P* = 0.14; retroperitoneal fat males, *P* = 0.0004, females, *P* = 0.002; *t*‐test).

To establish the effect of the HFHS diet on growth rate and fat deposition, body weight, total body fat mass and individual fat deposit masses were measured. A mixed effects analysis was performed to compare the effects of age and diet on body mass. With male body weight (Figure 4a), there were main effects of both age (*P* < 0.0001) and diet (*P* = 0.0005), with a significant interaction between the two factors (*F*_10, 303_ = 16.8, *P* < 0.0001). In the females (Figure 4b), there were also main effects of both age (*P* < 0.0001) and diet (*P* < 0.0001), with a significant interaction between the two in influencing body weight (*F*_10, 298_ = 40.3, *P* < 0.0001). The HFHS diet led to an increase in body weight in both sexes, which was significant by 11 weeks in the males and by 9 weeks in the females, relative to their respective controls. At 14 weeks, the total body fat mass (grams) measured by DEXA scanning (Figure 4c,d) was significantly greater in both the male (Figure 4e; *t* = 4.7, *P* = 0.005, Welch's t‐test) and female mice (Figure 4g, *t* = 8.7, *P* < 0.0001, Welch's *t*‐test) fed the HFHS diet compared with their control counterparts, at the expense of the lean mass (male: Figure 4f, *t* = 4.3, *P* = 0.001, Welch's *t*‐test; female: Figure 4h, *t* = 4.9, *P* < 0.0001, Welch's *t*‐test). The weights of the perirenal, retroperitoneal and gonadal fat deposits were also significantly increased in both males and females fed the HFHS diet relative to their controls (Figure 4i–p). In males fed the HFHS diet, the percentage contribution of these individual fat deposits to the greater total fat mass increased significantly for each of the three deposits (Figure 4n; perirenal fat, *P* = 0.02, gonadal fat, *P* = 0.002, retroperitoneal fat males, *P* = 0.0004; *t*‐test of arcsin transformed data). In contrast, in females fed the HFHS diet, only the retroperitoneal fat accounted for a significantly greater proportion of the increased total fat mass relative to mice fed with the control diet (Figure 4p; perirenal fat, *P* = 0.08, gonadal fat, *P* = 0.14, retroperitoneal fat males, *P* = 0.002, *t*‐test of arcsin transformed data). Fat accumulation, therefore, differed between males and females fed the HFHS diet, with the males gaining proportionally more visceral fat whereas the females appeared to increase their adiposity more generally.

### 3.3 Effect of HFHS diet on anxiety‐related behaviour in male and female mice

**FIGURE 5:**
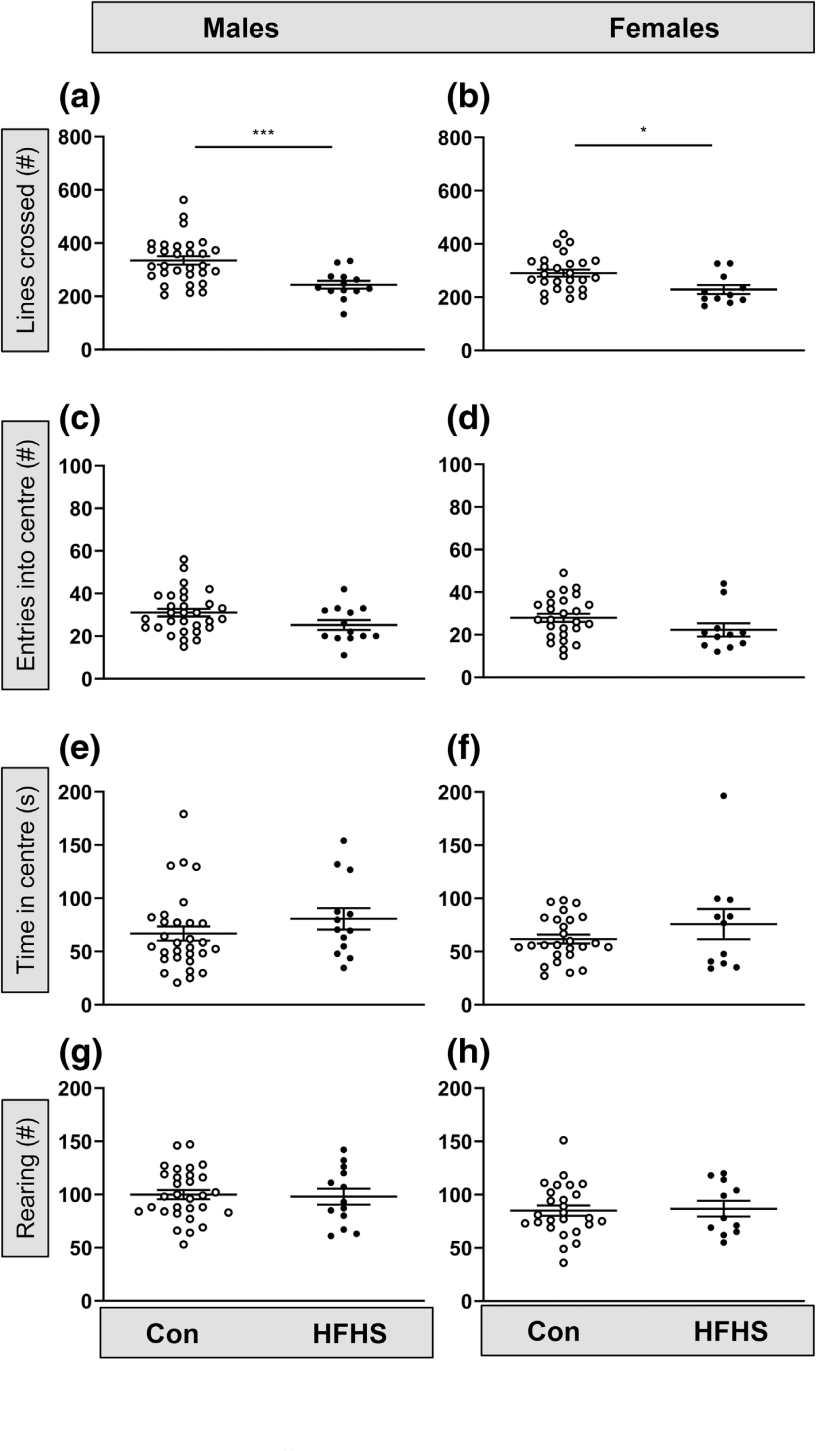
An obesogenic diet affects locomotor but not anxiety behaviour in the open field (OF). (a) The number of lines crossed in the OF by male mice (11–13 weeks) on a control or high fat–high sugar (HFHS) diet (***P = 0.001, *t*‐test). (b) Lines crossed by female mice (11–13 weeks) on a control or HFHS diet (*P = 0.01, Mann–Whitney). (c,d) Entries into the centre of the OF by 11–13 week old male (*P* = 0.06, *t*‐test) and female (*P* = 0.09, Mann–Whitney) mice on a control versus an HFHS diet. (e,f) Time (seconds) spent in the centre of the OF by 11–13 week old male (*P* = 0.17, Mann–Whitney) and female (*P* = 0.54, Mann–Whitney) mice on a control or HFHS diet. (g,h) Number of rears made in the OF by 11–13 week old male (*P* = 0.83, *t*‐test) and female (*P* = 0.85, *t*‐test) mice on a control or HFHS diet. Numbers of mice: control diet, 30 males and 26 females; HFHS diet, 13 males and 11 females.

Given the observed sex‐specific differences in fat distribution and in behaviour with sex and age, the effect of the HFHS diet on locomotor, anxiety‐related and exploratory behaviour was examined separately in the two sexes aged 11–13 weeks. In the OF test, there was a decrease in locomotion (number of lines crossed) in both sexes fed the HFHS diet compared with their control counterparts (males: *t* = 4.3, *P* = 0.0001, Welch's *t*‐test, Figure 5a; females: *P* = 0.01, Mann–Whitney, Figure 5b). In both males and females, anxiety‐related behaviour did not differ significantly with diet, either when measured as entries into the centre, (males: *t* = 1.9, *P* = 0.06, Welch's t‐test, Figure 5c; females: *P* = 0.09, Mann–Whitney, Figure 5d) or as duration in the centre (males: *P* = 0.17, Mann–Whitney, Figure 5e; females: *P* = 0.54, Mann–Whitney, Figure 5f). Exploratory rearing in the OF was also unaffected by diet in either male mice (Figure 5g, *t* = 0.2, *P* = 0.83, Welch's *t*‐test) or female mice (Figure 5h, *t* = 0.2, *P* = 0.85, Welch's *t*‐test). The data indicate there are no sex‐specific differences in the effect of diet on locomotion, anxiety‐related or exploratory behaviours in the OF task.

**FIGURE 6:**
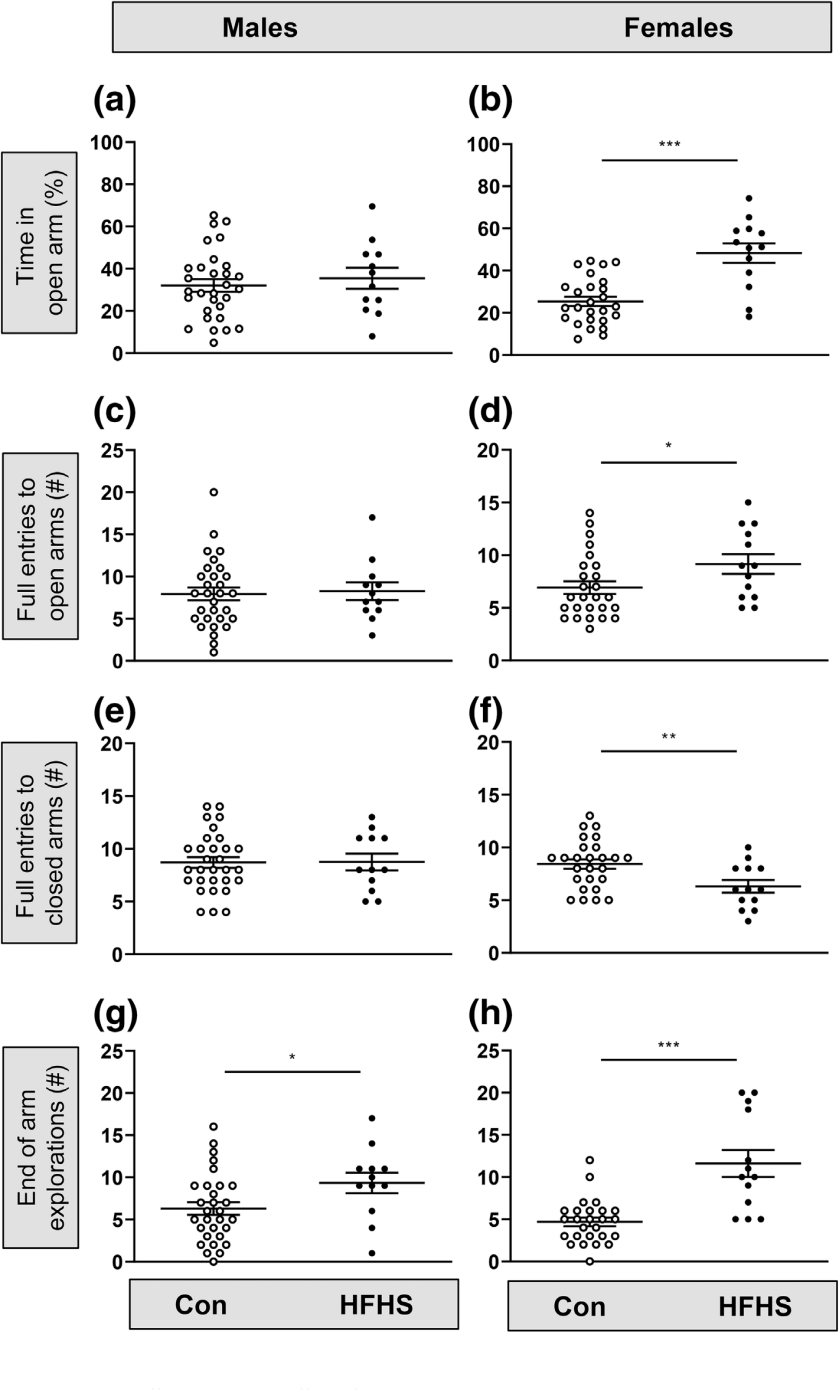
Sex differences in the effect of an obesogenic diet in the elevated plus maze (EPM). (a) The percentage time spent in the open arm of the EPM by male mice (11–13 weeks) on a control or high fat–high sugar (HFHS) diet (*P* = 0.57, *t*‐test). (b) Percentage time in the open arm for female mice (11–13 weeks) on a control or HFHS diet (***P = 0.0003, *t*‐test). (c,d) Number of full entries into the open arm by 11–13 week old male (*P* = 0.81, *t*‐test) and female (*P = 0.04, Mann–Whitney) mice on a control or HFHS diet. (e,f) Number of full entries into the closed arm by 11–13 week old male (*P* = 0.96, *t*‐test) and female (**P = 0.009, *t*‐test) mice on a control or HFHS diet. (g,h) Number of explorations to the end of the open arm by 11–13 week old male (*P = 0.048, *t*‐test) and female (***P < 0.0001, Mann–Whitney) mice on a control or HFHS diet. Numbers of mice: control diet, 30 males and 26 females; HFHS diet, 12 males and 14 females.

In the EPM task (Figure 6), there were significant effects of diet primarily in female mice. Relative to control mice, in females fed the HFHS diet, there were significant increases in the percentage time spent in the open arm (Figure 6b, *t* = 4.5, *P* = 0.0003, Welch's *t*‐test) and in the number of full entries into the open arm (Figure 6d, *P* = 0.04, Mann–Whitney), with a concomitant decrease in the number of full entries into the closed arm (Figure 6f, *t* = 2.8, *P* = 0.009, Welch's *t*‐test). Females fed the HFHS diet also made significantly more explorations to the end of the open arm (Figure 6h, *P* < 0.0001, Mann–Whitney). In male mice fed the HFHS diet, the only significant difference in behaviour observed in the EPM compared with mice fed the control diet was the increased number of explorations to the end of the open arm (Figure 6g, *t* = 2.1, *P* = 0.048, Welch's *t*‐test). The data indicate marked sex‐specific differences in the effect of an obesogenic diet on anxiety‐related behaviours in the EPM.

### 3.4 Effect of HFHS diet on social behaviour in male and female mice

**FIGURE 7:**
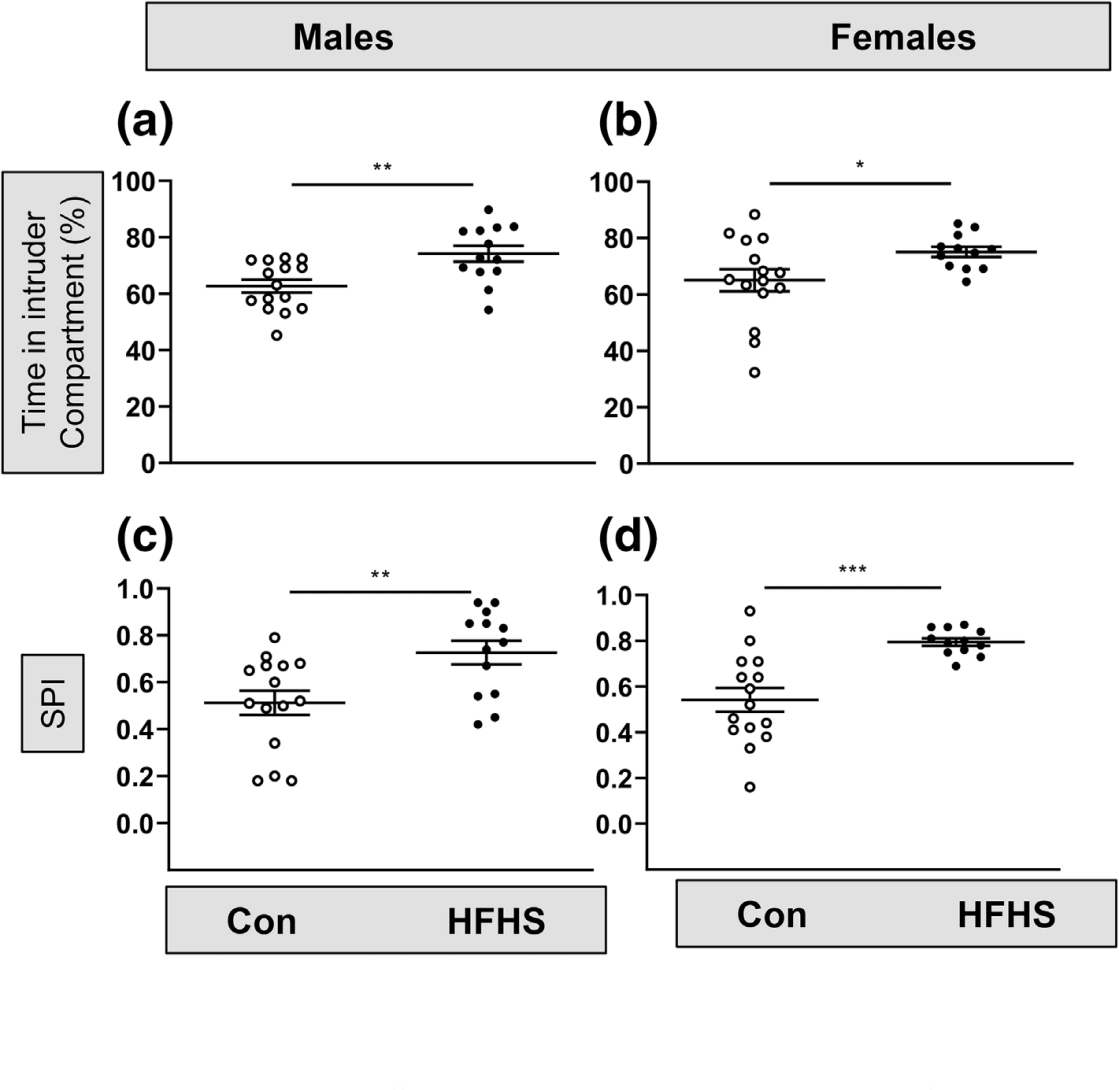
An obesogenic diet affects social interaction behaviour in male and female mice. (a) Male mice on the high fat–high sugar (HFHS) diet (*n* = 13) spent a significantly greater percentage time in the intruder compartment versus the object compartment than male mice on the control diet (*n* = 15; **P = 0.004, *t*‐test). (b) Female mice on the HFHS diet (*n* = 12) spent a significantly greater percentage time in the intruder compartment versus the object compartment than male mice on the control diet (*n* = 15; **P = 0.03, *t*‐test). (c) The social preference index, a measure of the time spent interacting with the intruder mouse versus the object, was significantly increased in male mice (*P* = 0.007, *t*‐test). (d) The social preference index was significantly increased in female mice (*P* = 0.0002, *t*‐test).

There was a significant effect of the HFHS diet on SP in both male and female mice. Compared with mice fed the control diet, both male and female mice fed the HFHS diet spent an increased percentage of time in the intruder mouse compartment relative to the TNS compartment (males: *t* = 3.2, *P* = 0.004, Welch's *t*‐test, Figure 7a; females: *t* = 2.3, *P* = 0.03, Welch's *t*‐test, Figure 7b). Both male and female mice fed the HFHS diet spent significantly more time interacting with the other mouse compared with the object, measured as the SP Index (males: *t* = 2.9, *P* = 0.007, Welch's *t*‐test, Figure 7c; females: *t* = 4.6, *P* = 0.0002, Welch's *t*‐test, Figure 7d). These data indicate that diet‐induced obesity significantly alters SP with the HFHS‐fed mice spending more time with a novel mouse over an object.

## 4 DISCUSSION

The data show that age and sex are contributing factors to the performance of control‐fed wild‐type C57BL6/J mice in a range of commonly used rodent behavioural tasks. These factors differentially affected behavioural outcomes during the transition from late adolescence to early adulthood, including locomotor activity and anxiety‐related behaviours. Consuming a diet high in fat and sugar reduced food and calorie intake, increased fat deposition and adiposity and resulted in sex‐specific effects on locomotion and anxiety‐related behaviours. Together, the findings demonstrate that age and sex are critical factors that need to be taken into account when designing behavioural studies in mice and in interpreting behavioural phenotypes that result from dietary and other environmental manipulations.

### 4.1 Effect of age and sex on locomotion and anxiety‐related behaviour

Although several studies have reported differences in various behaviours between young and aged animals (Lalonde & Strazielle, 2009; Macri et al., 2003; Nolte et al., 2019; Shoji et al., 2016), relatively little is known about the effect of age on behavioural changes at three key stages of the life course—from late adolescence to middle age. Using the OF and EPM behavioural tasks, the current study shows changes in multiple behavioural parameters over this period, including locomotion, exploration and anxiety‐related behaviours. Age significantly reduced locomotion within the OF arena, with significantly less movement at 20–22 weeks than earlier in life in both sexes. This result is consistent with previous rodent studies that have reported decreases in locomotion with progressing age through to adulthood (Davis et al., 2012; Shoji et al., 2016). In the current study, the reduction in locomotive activity appeared to be progressive with age in the females but later in onset in the males. In both sexes, this decline in activity may be explained by an age‐related decrease in muscular strength and motor function or reduced motivation to explore a novel, stressful environment. Mice are not considered aged at 20–22 weeks of age (Hagan, 2017); however, Shoji and colleagues (2016) propose that aging from young adulthood to middle age in mice may be associated with decreases in motivation to approach and investigate a novel social environment. Further studies are needed to establish whether the reduced locomotion with age is due to a decreased motivation to explore or to a true reduction in locomotive ability and the extent to which these factors are sex‐specific.

In addition to the locomotive changes, the older male mice showed a decrease in the number of entries into the centre of the OF arena and performed fewer rearings, both of which are indicative of anxiety‐related behaviours. In contrast, these age‐related changes were not seen in females. In the EPM, older male mice showed a significant decrease in closed arm entries and an increase in the time spent in the open arms, normally suggestive of anti‐anxiety behaviour. Whereas, female mice showed a decline in both the time spent in the anxiogenic open arm and the percentage of full entries into the open arm at 11–13 weeks of age, with a reduced number of open arm explorations at 20–22 weeks of age, which could be interpreted as an increase in anxiety‐like behaviour. The apparent contradiction in the results on anxiety from the OF and EPM tasks has been reported previously in several inbred and outbred mouse strains (Griebel et al., 2000; Hattori et al., 2012; Miyakawa et al., 2003; Takao et al., 2013). It has been proposed that a higher level of anxiety or high escape response to EPM exposure could manifest in higher levels of open arm exploration (Hattori et al., 2012; Holmes et al., 2000; Miyakawa et al., 2003; Takao et al., 2013). This behaviour has been reported in male mice in the EPM (Shoji et al., 2016), and rather than indicating a high level of anxiety, it may represent another approach of the mice, namely, to identify and explore the most likely route of escape via the open arms. This hypothesis is partially supported by the previous finding that aged animals have higher plasma corticosterone levels than younger animals after exposure to a novel environment or a sudden noise (Herman et al., 2001; Kucuk et al., 2008; Takao et al., 2013). Taken together, the current findings on locomotion or activity and exploratory‐ and anxiety‐related behaviours suggest that the two sexes adopt different behavioural strategies in response to novel, potentially stressful environments that depend on the specific nature of the challenge and the age of the mice.

### 4.2 Effect of a HFHS diet on food intake, growth rate, fat deposition and behaviour

There is a growing body of evidence from both human epidemiologic and animal experimental studies linking the consumption of diets high in fat or high in both fat and sugar with abnormal brain morphology and adverse neurodevelopmental and psychiatric morbidity (Contu & Hawkes, 2017; Edlow, 2017; Godfrey et al., 2017). In human longitudinal studies, it is difficult to disentangle the influence of the maternal environment from the postnatal environment, as the diet in childhood and adolescence is likely to reflect the diet of the mother during pregnancy. The available literature indicates a commonality between the behaviour influenced by maternal obesity and postnatal obesity. Few studies, however, have investigated the effects of an obesogenic diet provided from post‐weaning to adulthood. In the current study, post‐weaning consumption of an HFHS diet led to the expected increases in body mass and adiposity in both male and female mice, with a two to three fold increment in total body fat mass and an even greater sex‐specific percentage increase in the individual visceral fat deposits. These increases in body mass and fat accumulation occurred despite the mice consuming fewer kilocalories than their control‐fed counterparts. Previous studies of rodents fed obesogenic diets in which food intake was measured have shown both increases and decreases in daily kilocalorie consumption (Haleem & Mahmood, 2021; Wang et al., 2016; Zanini et al., 2017). Increased adiposity is likely to increase the expression of leptin, which, in turn, would act to decrease appetite and reduce food intake (Fried et al., 2000), consistent with the current finding of reduced food intake by the mice accumulating more fat on the HFHS diet. In turn, the reduced food and protein intake may account for the reduced lean mass observed in both the male and female mice fed the HFHS diet. With less lean mass, the mice fed the HFHS diet are likely to have a reduced metabolic rate and energy expenditure, which would contribute to a lower kilocalorie requirement. Future studies of the overall metabolic rate and the concentration of appetite and metabolism regulatory hormones are needed to understand how the HFHS diet leads to altered energy intake and body composition.

The consumption of an HFHS diet altered the behaviour in both male and female mice. In the OF task, both male and female mice showed reduced locomotion, providing evidence of lower innate activity in the fatter mice, which would contribute to a lower overall energy expenditure. A variety of outcomes have been reported regarding the locomotion or activity in diet‐induced obesity models. Studies that fed mice with a high fat diet or a high sucrose only diet have reported either no change or increased locomotion (Eudave et al., 2018; Sharma & Fulton, 2013). Reduced locomotion within the OF task has been reported in obese mice (Buchenauer et al., 2009; Takao et al., 2013), which is consistent with the current results. This reduction in locomotion may be due to the increased body mass of the mice, less lean muscle mass or reduced motivation to explore the surrounding novel environment. In people with a higher BMI, depression is more prevalent than in those in the healthy BMI range (Luppino et al., 2010; Strine et al., 2008). Moreover, depressive behaviours have been reported in mice fed a high fat diet (Yamada et al., 2011). In the current study, female mice fed the HFHS diet spent more time in the EPM open arms, suggesting they were comfortable exploring the normally anxiogenic open arm environment. Furthermore, both males and females fed the HFHS diet showed increased visits to the distal end of the open arm, suggesting either reduced anxiety or increased motivation. Diet‐induced postnatal obesity has been reported to induce anxiety in experimental animals in some, but not all studies using novelty‐suppressed feeding, OF tasks and elevated zero maze (Akter et al., 2020; Almeida‐Suhett et al., 2017; Bocarsly et al., 2015; Eudave et al., 2018; Xu et al., 2018; Zemdegs et al., 2016). Consistent with the current findings, rats and mice fed a high fat diet display reduced anxiety‐related behaviour within the OF and EPM tasks (Haleem & Mahmood, 2021; Xu et al., 2018). These published studies only used male animals, unlike the current study where reduced anxiety‐related behaviours were evident predominantly in female mice. Opposing anxiety behaviour outcomes within a published mouse obesity model have been reported (Cipriano et al., 2016) with increased anxiety‐related behaviours evident in the OF task, but not in the elevated zero maze (Eudave et al., 2018).

Few experimental studies have examined the effects of a post‐weaning diet high in fat and sugar on social behaviour, as the majority of the research has primarily focused on the effects of maternal obesity on their adult offspring. In the current study, mice fed the HFHS diet post‐weaning showed an increased preference for interacting with a novel mouse rather than an object. This effect was evident in both sexes. A previous study has reported that a short, 10‐day exposure to a high fat or high sucrose diet did not alter social behaviour (Eudave et al., 2018). However, a longer period of 8 weeks on a high fat diet led to an increased social interaction in male rats (Buchenauer et al., 2009), which supports our finding of increased social interaction after 8–9 weeks on the HFHS diet. In the current study, the increase in sociability observed in the SP task combined with the increased number of visits to the distal end of the open arm of the EPM may be indicative of an increased risk‐taking or novelty‐seeking behaviour (Ludwig et al., 2021; Shoji & Miyakawa, 2021). Further studies are therefore needed to establish the true cause of this apparent increased sociability in the mice fed the HFHS diet. In summary, in the current study, the post‐weaning consumption of an obesogenic diet high in both fat and sugar led to a significant reduction in locomotion or activity in both sexes, differential effects on anxiety‐related behaviours to height and open spaces in females only and significantly increased SP in both male and female mice. This data shows that diet‐induced obesity exclusively in postnatal life leads to altered behaviours in both males and females in a sex‐specific manner.

## 5 CONCLUSIONS

The current results show that aspects of mouse behaviour are sex‐specific. The effects of age and feeding an obesogenic diet on locomotion and/or anxiety behaviours depended on the sex of the mouse. Males and females appeared to adopt different behavioural strategies in coping with novel, stressful conditions. These responses also differed depending on their diet and on whether the anxiety was induced by the height of the EPM or the open space within the OF arena. Overall, the findings highlight the role of age, diet, food intake and body composition of the mice on behaviour. The results also emphasise the importance of including both sexes and of careful consideration of age in behavioural studies.

## AUTHOR CONTRIBUTIONS

**Emily J. Camm**: Conceptualisation; data curation; formal analysis; funding acquisition; investigation; methodology; project administration; resources; supervision; validation; writing—original draft; writing—review and editing. **Abigail L. Fowden**: Conceptualisation; formal analysis; methodology; project administration; resources; supervision; validation; writing—review and editing. **Emily J. Mort**: Data curation; formal analysis; funding acquisition; investigation; validation; visualisation; writing—review and editing. **Susan Jones**: Formal analysis; project administration; supervision; validation; visualisation; writing—original draft; writing—review and editing. **Surina Fordington**: Investigation; writing—review and editing. **Sophie Heritage**: Investigation; writing—review and editing.

## CONFLICT OF INTEREST STATEMENT

The authors declare no conflicts of interest.

### PEER REVIEW

The peer review history for this article is available at https://www.webofscience.com/api/gateway/wos/peer-review/10.1111/ejn.16070.

## Data Availability

Data have been uploaded to Apollo, the University of Cambridge repository and to Dryad (https://datadryad.org/stash/share/ef2blxA6aZJoAdiahp6q6G0bGzm0_gTSJZfSOIz_G94). [Correction added on 14 July 2023, after first online publication: The Data Availability Statement has been updated in this version.]
